# Roles of Social Protection to Promote Health Service Coverage among Vulnerable People toward Achieving Universal Health Coverage: A Literature Review of International Organizations

**DOI:** 10.3390/ijerph20095754

**Published:** 2023-05-08

**Authors:** Yuta Yokobori, Hiroyuki Kiyohara, Nadila Mulati, Kaung Suu Lwin, Truong Quy Quoc Bao, Myo Nyein Aung, Motoyuki Yuasa, Masami Fujita

**Affiliations:** 1 Bureau of International Health Cooperation, National Center for Global Health and Medicine, Tokyo 162-8655, Japan; 2 Department of Global Health Research, Graduate School of Medicine, Juntendo University, Tokyo 113-8421, Japan; 3 Department of Global Health Policy, Graduate School of Medicine, The University of Tokyo, Tokyo 113-8654, Japan; 4 Institute for Community Health Research, University of Medicine and Pharmacy, Hue University, Hue 530000, Vietnam

**Keywords:** social protection, social assistance, vulnerable population, UHC

## Abstract

A wider range of social protection services, including social insurance and social assistance, are gaining global attention as a key driver of improved health service coverage and financial protection among vulnerable populations. However, only a few studies have investigated the associations between social protection and universal health coverage (UHC). Therefore, we conducted a literature review on relevant international organizations with respect to this topic. We found that many international organizations consider the wide range of social protection services, including social insurance and social assistance, essential for achieving UHC in 2030. In specific health programs, social protection is considered an important service to promote health service access and financial protection, especially among vulnerable populations. However, discussions about social protection for achieving UHC are not given high priority in the World Health Organization. Currently, the coverage of social protection services is low among vulnerable populations in low- and middle-income countries. To address this issue, we employed the metrics recommended by the migrant integration policy index (MIPEX). Based on our findings, a conceptual framework was developed. We expect this framework to lead synergy between social protection and health systems around the globe, resulting in healthy ageing.

## 1. Introduction

Universal Health Coverage (UHC) by 2030 is one of the targets of the Sustainable Development Goals adopted in 2015 [1] and stated in the political declaration of the high-level meeting on UHC in 2019 [2]. However, according to the 2019 Global Monitoring Report [3], while the UHC Service Coverage Index has improved globally from a population-weighted average of 45 in 2000 to 68 in 2019, progress is slow in low-income countries. In addition, the population facing catastrophic or impoverished health spending was still estimated to be between 1.4 and 1.9 billion in 2017.

The progress towards UHC has been delayed among vulnerable populations in low- and middle-income countries. The global monitoring report highlights the persistent inequalities across households within countries and calls for the need to improve health service coverage and prevent catastrophic expenditure for vulnerable populations. Sufficient health coverage of vulnerable populations would minimize the impact of health crises in a country. For example, during the COVID-19 pandemic, vulnerable populations had the highest risk of infection and were severely affected in terms of health as well as economic and social status [4,5]. Without effective infection control services coverage, these populations could act as incubators for further outbreaks nationwide [6,7]. Therefore, it is important to focus on vulnerable populations to accelerate the progress towards UHC and improve disease control in future health crises.

In order to ensure financial protection for UHC, health insurance schemes have received global attention [8] as a means to reduce inequities between population groups [9]. However, current strategies in low- and middle-income countries have not achieved their aims [10] due to difficulties associated with identifying the most vulnerable populations [11,12,13] and the socioeconomic factors that impede access to basic health services [14]. In order to address issues related to poverty in a holistic manner, multisectoral approaches should be considered. Several international organizations, such as the United Nations (UN), the International Labor Organization (ILO), and the World Bank, are beginning to discuss the need to develop not only health insurance but also a broad range of other social protection services such as social assistance across sectors to better promote and deliver UHC for vulnerable populations [15,16]. However, different international organizations have different positions about the roles of social protections under the context of UHC.

Nevertheless, few studies have illustrated the associations of social protection, especially services other than social insurance, with UHC from a global perspective. Accordingly, this review has the following objectives: 1. To investigate the associations between different types of social protection services and health, including social insurance and social assistance, in terms of vulnerable individuals. 2. To review existing responses to health issues by international organizations in terms of social protection. 3. To review the positions of different international entities on the associations between social protection and UHC. 4. Finally, to analyze the global discussions in health sectors (e.g., World Health Organization (WHO)) about social protection and UHC.

## 2. Materials and Methods

### 2.1. Search Strategy

**Figure 1:**
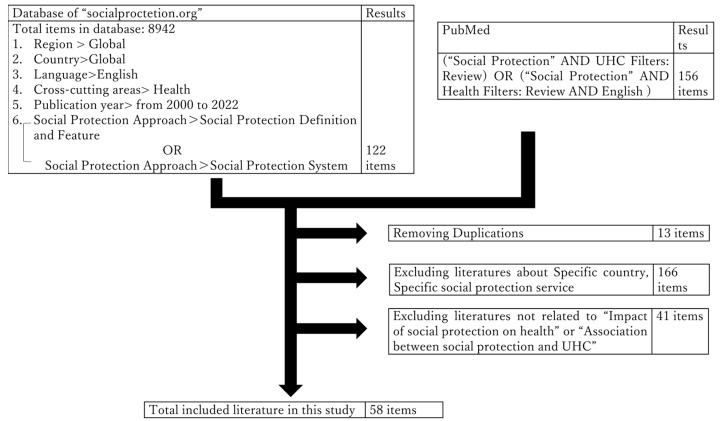
Search strategy.

We searched the literature on the associations between social protection and UHC using PubMed for English-language articles published before September 2022. The search strategy followed the Preferred Reporting Items for Systematic Reviews and Meta-Analyses (PRISMA) guidelines and was performed using the following search terms: “social protection” and “UHC” or “social protection” and “Health”. In order to capture information from international entities, we searched the publications by international organization through the database “socialprotection.org” at the address https://socialprotection.org/discover/publications, access on 1 October 2022. “socialprotection.org” is an online member-based knowledge-sharing and capacity-building platform established in 2015 to respond to a recommendation from the G20 Development Working Group. The database “socialprotection.org” provides the most up-to-date and comprehensive content on social protection, with a focus on low- and middle-income countries. The detailed search strategy employed on “socialprotection.org” is shown in Figure 1. In order to follow the global discussions about social protection and UHC in health sectors, we searched all WHO resolutions and decisions in the world health assembly for the past 10 years from 2013 to 2021 [17,18,19,20,21,22,23,24,25,26,27] and the main documents of agenda with UHC in the title.

### 2.2. Study Selection and Synthesis Strategy

Out of the extracted publications from PubMed and “socialprotection.org”, we removed duplicates and excluded any literature that focused on specific countries or specific vulnerable populations because this review aimed to capture an overview of global movements of social protection as a whole. We included publications that explained the impact of social protection in relation to health and UHC. The three co-authors independently collected and screened the data from selected studies, and the corresponding author supervised their work and sorted the extracted data according to the two perspectives noted above: “impact on health” by different types of social protection services in different settings and “association with UHC” proposed by different international organizations. Regarding the WHO resolutions and decisions, we investigated the occurrences of the terms “social protection”, “social assistance”, or “cash transfer” within UHC-related agendas. In addition, we examined the occurrence of the term, “social protection” in the documents of agendas and “UHC” in the titles. After the selection process, we summarized the findings according to our four research objectives. As the synthesis, the extracted data was sorted according to four objectives stated at the end of the introduction section. In order to review the evidence of associations between social protection and health, the academic articles extracted through the search strategy were investigated. Regarding the other objectives, the publications from relevant international organizations were mainly examined.

## 3. Results

As described in Figure 1, 122 and 156 items were collected from searching “socialprotection.org” and PubMed. Among these publications, 58 items (28–85) are included in this literature review. The items comprise fourteen academic papers, five reports from Thinktanks (Institute of Development Studies, the Overseas Development Institute ODI and European University Institute) and thirty-nine publications from international organizations: thirteen from the World Bank, nine from the ILO, six from the UN, four from the United Nations Children’s Fund (UNICEF), three from the European Union (EU), two from the Organization of Economic Co-operation and Development (OECD), and two from the World Health Organization (WHO). 

### 3.1. Evidence of Associations between Social Protection and Health among Vulenerable Populations

According to the definition of social protection and discussions about its relationship with UHC, social protection comprises at least social insurance and social assistance as common elements towards achieving UHC. Both elements are defined by the UN [28] as “Social Insurance”, which is a program providing protection against life-course contingencies such as maternity and old age, or work-related contingencies such as unemployment or sickness and “Social assistance”, which is program providing support for those in poverty. Normally, social insurance is financed from contributions by workers and their employers, whereas social assistance is financed through taxation. This section summarizes the evidence regarding these social protection services in terms of health service utilizations and financial protection, especially among vulnerable populations. 

#### 3.1.1. Social Insurance

Several systematic reviews have shown the positive impacts of social insurance. As Comfort et al. suggest, the provision of social insurance is associated with increased maternal and child health service utilization [29,30]. Spaan et al. found the positive effects for health service utilization and financial protection [30,31]. In contrast, Acharya et al. (2012) suggested weak evidence of the effects on fiscal protection, especially for the poorest populations [30,32]. Meanwhile, a review of 19 papers by the WB found no evidence of the impact on health service utilization or fiscal protection. However, it does report on the effects of some health insurance, but the effects are minimal for the poorest populations [30,32]. In summary, while social insurance appears to be effective in improving health service utilization and financial protection, some studies maintain that the impact on vulnerable populations is limited.

#### 3.1.2. Social Assistance

Social assistance includes several types of services that the WB categorizes under five headings: cash transfers, non-cash transfers, short-term employment, income generation, capacity building and institutional improvements [33,34,35]. This section focuses on the impacts of cash transfers as an exemplary representative of social assistance. Adato et al. suggested that cash transfers have impacts on health in the following ways [36]: (1) compensating for direct costs of accessing health care (transportation, medical costs, and opportunity costs), (2) improving nutrition status by higher quality diet as well as a larger quantity of food, and (3) providing incentives for people to participate in prevention and health education activities by conditional cash transfer. The authors reviewed 30 cash transfer programs, including 20 conditional cash transfers and 10 unconditional cash transfers, and found that cash transfer programs in Africa and Latin America could have positive effects on health service utilization. Pointedly, conditional cash transfers in Mexico could even reduce child mortality. 

Bastagli et al. also reviewed 200 studies on cash transfer and concluded that most of the studies showed an improvement in health service utilization and that there was also an improvement in growth rates [37]. In addition, Hunter et al. reviewed 51 studies in which cash transfer led to improvements in the utilization of maternal and child health-related services [38]. Glassman et al. also found that conditional cash transfer improved maternal and child health indicators and reduced the incidence of low birth weight [39]. Finally, Owusu (2018) conducted a qualitative review of cash transfer, focusing on sub-Saharan Africa, and reported that it helped improve health service utilization and financial protection among vulnerable population [40]. In summary, most of the results obtained by systematic review and qualitative study showed that social assistance had a positive effect on health service utilization and health outcomes, especially among vulnerable populations.

### 3.2. Responses to Health Issues by International Organizations through Social Protection

Since many studies emphasize the role of social protection to improve health status in cases of specific diseases or conditions, we reviewed the existing responses identified by the selected publications, including those related to HIV/AIDS, tuberculosis, and COVID-19, as well as humanitarian settings.

#### 3.2.1. Control of Tuberculosis and HIV/AIDS

The relationship between tuberculosis control and social protection is discussed by the WHO Western Pacific Regional Office (WPRO) [41,42]. They state that the patients’ loss of income related to tuberculosis is the greatest risk for treatment interruption and, thereby, social protection should include support for non-medical expenditure. Income security, through social protection, can make contributions to the successful control of tuberculosis among vulnerable populations. UNICEF states that social protection can mitigate the socioeconomic impact of HIV/AIDS by addressing social determinants of health, leading to the mitigation of barriers to accessing HIV/AIDS services [43]. Furthermore, the OECD suggests that social protection has the potential to effect social change and enable women to access their rights and claim entitlement among HIV/AIDS patients [44]. Toska et al. also mention that social protection can mitigate socioeconomic risks and break the cycle of HIV infection in children born to infected parents. In particular, cash transfers linked to definite care is effective for vulnerable populations [45]. Finally, Van der Wal et al. [46], state that broad support for livelihood and work can improve the socioeconomic status of patients and contribute to the improvement of HIV/AIDS-related indicators. Likewise, the literature related to tuberculosis and HIV/AIDS emphasizes the importance of wide ranges of social protection for infection control, especially among vulnerable populations.

#### 3.2.2. COVID-19 Pandemic

Since the COVID-19 pandemic, many international organizations have emphasized the importance of social protection to promote health service access. Firstly, the UN’s framework for the immediate socioeconomic response to COVID-19 incorporates social protection and basic service into one of the five pillars [47,48,49]. To mitigate the impact of COVID-19 on vulnerable populations, the UN suggests that governments need to expand to a wider range of social protection, including cash transfers, food assistance, and social insurance. Furthermore, social protection is considered an essential mechanism to provide access to health services and to protect against financial crises associated with unemployment and sick care. The WPRO also acknowledges that WHO recommendations on COVID-19 measures are not realistic for people who cannot access health services due to difficult circumstances and limited resources. 

Therefore, social protection services facilitated through cash transfers and in-kind support are critical to remove the barriers to health facilities and ensure the protection of these populations [50]. These perspectives are supported by the WB, which states that social protection can respond to demand on the beneficiary side and promotes health service access relating to COVID-19 [5]. Similarly, the International Labour Organization (ILO) highlights the importance of basic health services and income guarantees from the perspective of the Social Protection Floor [51]. Moreover, UNICEF notes that social protection can contribute to reducing the risk factors and strengthening the protective factors related to child protection issues. By assisting caregivers, services can improve the utilization of health services for children and mothers [52]. Lastly, the EU describes that social determinants of health need to be addressed through social protection to ensure access to health services for vulnerable populations that are most affected during crises [53]. Other than these official publications from international organizations, many academic papers also stress the importance of broad social protection services, including social assistance for the delivery of COVID-19 measures [54,55].

#### 3.2.3. Humanitarian Setting

Recent discussions suggest expanding the roles of social protections enacted during a health crisis to a broader range of humanitarian settings. WHO suggests that cash transfers are effective in reducing direct and indirect financial barriers and improving access to health services in a humanitarian context, especially for vulnerable populations [56]. The United Nations Office for the Coordination of Humanitarian Affairs (UNOCHA) also makes similar arguments [57,58]. Moreover, UNICEF proposes shock-responsive social protection, which is important to have during a crisis [59]. The WB also states that social protection, including cash transfers, is effective in improving health service access during natural disasters [60,61]. In addition, beyond emergency rescues, the EU proposes that social protection should be leveraged as the nexus between humanitarian crisis and development in societies [56]. In this context, social protection can be the point of contact between two key global agendas: health security and UHC. 

### 3.3. Positions of Different International Entities on Social Protection in the Context of UHC

According to Devereux et al., there is no consistent definition of social protection [62]. However, three main concepts underlie the definitions of the UN, ILO, the WB, and the EU/OECD [63]. Based on these definitions, each international organization discusses the associations between social protection and UHC in their publications. 

#### 3.3.1. UN/ILO

ILO defines social protection as the set of policies and programs designed to reduce and prevent poverty and vulnerability throughout people’s entire life cycle [64]. Following the tradition of the ILO, some authors advocate that social protection should be delivered to everyone as a human right. The rights-based approach considers citizens as “rights-holders” and states as “duty-bearers”. From this perspective, social protection can be seen as a development of social rights, such as equality, inclusion, and non-discrimination [63]. The ILO proposed the idea of “social protection floor”, which was launched in 2009 under the UN-led initiative from a human rights perspective [65,66]. In 2012, the UN issued guidance on basic social protection services tailored to the courses of peoples’ lives, including services to cover against the financial consequences of maternity, sickness, unemployment, work injury, invalidity, families with children, old age, and medical care [67,68]. These services should be complementary to each other [67] and covered by either a contributory (e.g., social insurance) or non-contributory (e.g., tax-funded social assistance schemes) [69]. 

The objective of ILO’s social protection policy in health is to provide access to basic health services to all those in need, and as part of the “social protection floor” and “universal social protection” concepts [70]. In this context, the ILO considers UHC as a part of the conditions that needs to be addressed by the “social protection floor [71]”. Furthermore, one report states that while moving towards achieving UHC, it is necessary to address the loss of income due to medical treatment by (non-contributory means) in addition to health insurance (contributory) [72,73]. The ILO also proposes the concept of social health protection [74] as an integral component of comprehensive social protection systems. Social health protection is a series of public or publicly organized and mandated private measures that aim to achieve effective access to quality healthcare without hardship and income security to compensate for lost earnings in case of sickness and to improve population coverage of social health protection measures, including improving legal coverage and raising awareness of entitlements and effective protection. Improving linkages and better coordination between access to medical care and income security are urgent needs to address key determinants of health [75]. The ILO concludes that social health protection is central to achieving the objective of UHC, which emphasizes the importance of financial protection and effective access to healthcare services [75].

#### 3.3.2. World Bank

The WB defines social protection as the systems that help the poor and vulnerable cope with crises and shocks, find jobs, invest in the health and education of their children, and protect the aging population; further, it explains social protection within the framework of Social Risk Management (SRM) to address hardships in people’s lives [76]. SRM is a conceptual framework developed for the WB’s Social Security Strategy 2001 [77] and has been repeatedly updated to SRN 2.0 in 2019 [76]. The WB suggests that social protection is the best answer to poverty alleviation, and allows the vulnerable to invest and accumulate assets, and, consequently, escape poverty [63]. The WB describes UHC as a part of the broad social protection policies, which are essential to meet the goals of SRM2.0 [76]. They also categorize social protection services into three components: social assistance (non-contributory), social insurance (contributory), and labor market programs [33,78,79,80]. Social assistance is designed to reduce poverty and inequality, and includes both conditional and unconditional cash transfer, food and in-kind transfers, school feeding programs, fee waivers, and targeted subsidies. Social insurance ensures adequate living standards amidst sudden life changes and includes contributory old-age, survivor and disability pensions, maternity or paternity benefits, and health insurance coverage.

#### 3.3.3. EU/OECD

The EU and OECD consider social protection as an efficient factor in pro-poor economic growth [63]. By adopting the social protection measures proposed by Devereux et al., the EU, and OECD propose that the roles of social protection can be sorted into four categories: protective, preventive, promotive, and transformative functions, in terms of how they progress the reduction of the vulnerability of the poor [81]. Ji-Yeun Rim et al. discussed the association of social protection with UHC [82] in the context of this framework: (1) Protective measures in income and in-kind transfers alleviate financial and material hardship experienced during illness and can complement the existing healthcare cost subsidies through social assistance services. (2) Preventive measures can prevent negative health impacts through wider social insurance. (3) Promotive measures can improve human capability by contributing towards livelihood assets and empowerment through education, training, and employment. These interventions can facilitate the access and utilization of health services through increased financial, human, and social capital. (4) Transformative measures promoting social inclusion and women’s empowerment can have a metamorphosing power, which can contribute towards barriers to access and utilization. This may include empowering trade unions to provide a voice for the vulnerable, launching public awareness campaigns seeking to change broader attitudes within the society, or implementing policies that specifically empower women.

However, many similar organizational frameworks exist, most of which are complementary [83]. Currently, there is an increasing consensus about the key elements of social protection [76], including the need to proactively protect individuals from present and future poverty. The main target group is the poor and/or those vulnerable to poverty. Increased attention is being devoted to the lifecycle of an individual or household as well as addressing both the “fairness” of outcomes and making opportunities less unequal. Regarding the association between social protection and UHC, most organizations suggest that the broad range of social protection services including social assistance are necessary to accelerate the progress of UHC, especially among vulnerable populations.

### 3.4. Global Discussions in the Health Sector about Social Protection and UHC

There are a few articles that mention social protection in the context of UHC. We found some publications that discuss about social protection as the measures against specific diseases, including COVID-19 [5] and tuberculosis [41,42], as described in Section 3.1. However, no other publications by the WHO noted social protection within the literature identified in the course of our search. As the results of reviews of resolutions and decisions, annexes in the World Health Assembly (WHA), several documents contain the term “social protection”. As described in Table 1, a wide range of documents came from vertical programs regarding issues such as tuberculosis, non-communicable diseases, and COVID-19 to cross-sectional programs such as social determinants of health, human resources, and refugees/migrants. Regarding social assistance, cash transfer is mentioned in some specific programs, such as vaccine, non-communicable diseases, nutrition, tuberculosis, and disability. However, no resolutions contain “social assistance” terms from 2016. While we found that twelve agendas and seven resolutions contained the term “universal health coverage” in the title, the main documents and the resolutions/decisions do not mention any “social protection” term except for the agenda, “17.3 Universal Health Coverage”, in WHA66 (Table 2 and Table 3). This agenda recognizes the request by a previous WHA resolution for a report of progress towards UHC, particularly with regard to equitable and sustainable health financing and social protection of health [84]. In summary, while social protection is mentioned in a broad range of resolutions and the annex in WHA, the discussions about social assistance are limited to some vertical programs regarding COVID-19 and TB. In addition, there is almost no discussion about the need for linkage and cooperation with social protection under the UHC agenda and related resolutions/decisions in the previous WHA, except for reporting the progress toward UHC.

## 4. Discussion

Several publications state that, although the effectiveness of social insurance is limited for vulnerable populations, social assistance has been reported to be effective in improving health service utilization, including for vulnerable populations. Social assistance is also reported to be important in times of crisis, not only to protect against livelihood impacts but also to promote health service utilization. This suggests that social assistance programs may be effective in promoting UHC, especially among vulnerable populations. However, there is no consistent definition of social protection; there is a common view that it is a proactive service against present and future poverty or hardship and comprises social insurance and social assistance. Towards achieving UHC, the UN, ILO, and WB have discussed the importance of social protection not only limited to social insurance but also social assistance. However, the WHO has published fewer reports and governance documents on the association between a broad range of social protection services and UHC while the discussions seemed to start with the topics of tuberculosis, HIV/AIDS, and COVID-19. This finding implies that the importance of comprehensive social protection and its linkage with health would be unlikely to be reflected in guidance for countries to formulate health policies being aimed toward UHC, beyond reporting the progress of UHC.

The coverage of social protection is currently low, especially in low- and middle-income countries. According to the report of WB [82] in 2018, social insurance programs are more prevalent in high-income countries, covering 60% of the poorest quintile. By contrast, in low-income countries, only 2% of the poorest quintile is covered by this type of program. Social assistance programs account for the most social protection program coverage of the poor in all of a respective country. However, there are gaps in coverage related to the countries’ specific contexts, as high-income countries report the highest coverage of poor people through social assistance programs (76%), compared with only 18% in low-income countries. The UN explains that the reasons for the low coverage of social protection in low-income countries may include: (1) many informal workers who are not registered in the civil registration system, (2) low capacity of advocacy and lack of information about available social protection services, (3) low awareness due to low socioeconomic status, (4) large distance between their residential areas and service points, (5) lack of money for the transportation and other necessary costs for procedures, and (6) discrimination of vulnerable population leads to exclusion from entitlement and unfriendly services in service points [85]. These factors can be obstacles to the utilization of services, especially among vulnerable populations.

**Figure 2:**
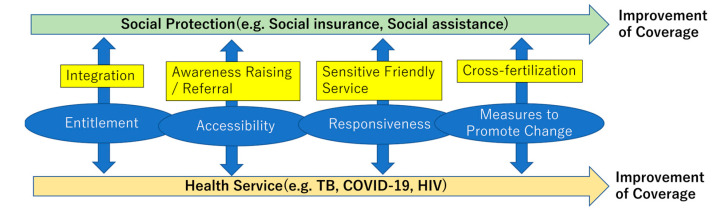
Synergy between social protection and health services for vulnerable populations.

In light of these findings, several measures should be considered to improve the coverage of social protection, including social assistance and health services for vulnerable populations. We categorized these measures using the framework of the Migrant Integration Policy Index (MIPEX) Health Strand for migrants [86], as they are considered a vulnerable population. The MIPEX Health Strand is an instrument developed under the support of the EU to measure the equitability of policies relating to the health of migrants in terms of four challenges: (A) entitlements to health services; (B) accessibility of health services, (C) responsiveness to people’s needs, and (D) measures to promote change. We adopted this framework because it encompasses critical factors that affect access to social protection and health services not only for migrants but also for other vulnerable populations. Furthermore, this framework could help identify points for synergies between social protection and health services, as illustrated in Figure 2. 

Regarding entitlement, social protection and health systems should be appropriately designed to ensure the entitlement for people in need, including targeting, registration, contents of benefits and services, and the necessary resource allocations. The partial integration of these measures between social protection and health services may contribute to the improved coverage of both. In the long run, a well-functioning civil registration system should be established to promptly identify expected recipients of services. Concerning accessibility, information on entitlements to benefits and services should reach vulnerable populations in a way they can comprehend. In order to address a range of barriers, including social, cultural, language, administrative, and navigators, or cultural mediators, such as peer workers, community leaders, volunteers, and consultation services, could play a major role in improving awareness among the populations and referring them to necessary service delivery points. These navigators or cultural mediators should be able to improve access not only to a single service but also a variety of services across social protection and health services, maximizing the opportunity. At the point of services, the responsiveness to the needs of recipients should be considered for interventions. As vulnerable populations tend to have complex psychosocial problems, including traumatic experiences using services, service delivery should be designed to be sensitive and friendly for vulnerable populations. The experiences of a single program in delivering vulnerable population-sensitive and friendly services should be shared and replicated across social protection and health services. Lastly, measures to promote changes include involving vulnerable populations in program management, monitoring and evaluation, research and advocacy, multi-sectoral coordination at central and local levels, and solidarity including social and solidarity economy (SSE) [87]. Advancement in each of these measures should be cross-fertilized across social protection and health services to maximize the expected outcomes, including coverage. Some of these synergies for vulnerable populations are observed in addressing specific health issues, such as tuberculosis, HIV, and COVID-19. Towards achieving UHC, these experiences should be replicated and expanded to address a wide range of health needs in a comprehensive manner.

**Figure 3:**
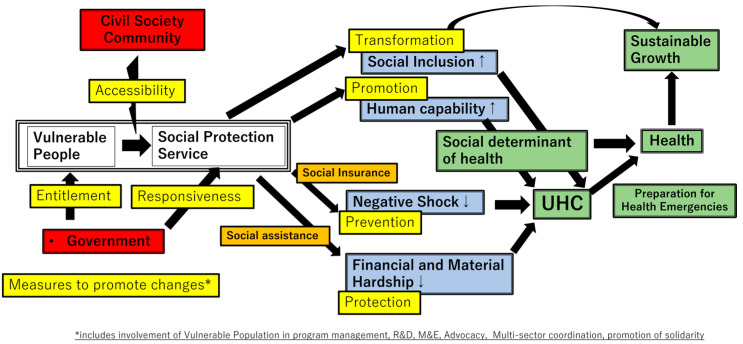
Association of social protection with UHC and key factors to promote social protection coverage.

Considering these findings, we developed a diagram to describe the association of social protection with UHC and the key factors to promote social protection coverage (Figure 3). Governments and donors should play a principal role for the “entitlement” to the social protections of people in need. It involves design of social protection systems, including registration, targeting, and types of services. They can also implement “measures to promote changes” to expand the coverage of quality social protection services. Civil society or communities could enhance support to vulnerable populations to improve “accessibility” to the points of service, which should be friendly and provided without any discriminations against them (“responsiveness”). We adopted the EU model: prevention, protection, promotion, and transformation, as a viable pathway from social protection to UHC because this model is comprehensive and explains the roles of social protection during emergency situations as well as development phases. Since social determinants of health (SDH) should be central to both the equitable pursuit of healthy lives and the provision of health services for all [88], we believe that the perspectives of promotion and transformation could have a great influence on the SDH in our model. This model has an advantage in showing the possible measures to improve social protection services and the objectives towards achieving UHC by a single diagram. 

Naturally, this study has some limitations. First, we did not analyze the association between social protection in specific vulnerable populations such as immigrants, the homeless, and the elderly. Since different populations may have different issues, further studies designed to specifically include these groups should be considered in the future. In addition, unique characteristics such as cultural backgrounds at the regional or country level should be taken into account. Therefore, reviews at the regional or country level will also be important to develop effective and efficient social protection services both across and within nations. Finally, while this literature review focuses on the framework to explain the relationship between social protection and UHC, the actual situations cannot be evaluated from the viewpoint of this framework. Further investigation is necessary to understand the challenges and functions of social protection services on the ground by field surveys at the level of individual countries.

## 5. Conclusions

Many international organizations note that a wide range of social protection measures, including social insurance as well as social assistance, are essential to accelerate the progress towards achieving UHC in 2030 from the viewpoints of protection, prevention, promotion, and transformation. As the social protection coverage has been increasing even in low- and middle-income countries, it is getting critical to promote cooperation and synergy between health and social protection including social assistance specifically for vulnerable populations, with particular to entitlement, accessibility and responsiveness of services, as well as measures to promote change. 

## Figures and Tables

**Table 1:** Occurrences of words of social protection and social assistance in WHO resolutions and decisions.

WHA	Year	Number	Title	SP *	SA *
WHA65	2012	WHA65.4	The global burden of mental disorders, and the need for a comprehensive, coordinated response from health and social sectors at the country level	2	0
		WHA65.8	Outcome of the World Conference on Social Determinants of Health	1	0
		ANNEX 2	Comprehensive implementation plan on maternal, infant and young child nutrition	5	2
		ANNEX 4	Global vaccine action plan	1	2
WHA66	2013	WHA66.9	Disability	1	0
		ANNEX 3	Comprehensive mental health action plan 2013–2020	3	0
		ANNEX 4	Global action plan for the prevention and control of noncommunicable diseases 2013–2020	4	1
WHA67	2014	WHA67.1	Global strategy and targets for tuberculosis prevention, care and control after 2015	1	0
		WHA67.20	Regulatory system strengthening for medical products	1	0
		ANNEX 1	Global strategy and targets for tuberculosis prevention, care and control after 2015	17	1
		ANNEX 3	WHO global disability action plan 2014–2021: better health for all people with disability	6	3
WHA68	2015	ANNEX 6	Outcome of the second international conference on nutrition	6	1
WHA69	2016	WHA69.11	Health in the 2030 agenda for sustainable development	1	0
		ANNEX1	Global strategy and action plan on ageing and health 2016–2020: towards a world in which everyone can live a long and healthy life	5	0
		ANNEX7	Global strategy on human resources for health: workforce 2030	1	0
		ANNEX8	Global health sector strategies on HIV, viral hepatitis and sexual transmitted infections, for the period 2016–2021	1	0
WHA70	2018	ANNEX 2	Working for health. Five-year plan for health employment and inclusive economic growth (2017–2021)	4	0
		ANNEX 4	Framework of priorities and guiding principles to promote the health of refugees and migrants	2	0
WHA71	2019	ANNEX 1	WHO Global Conference on noncommunicale diseases pursuing policy coherence to achieve SDG target 3.4 on NCDs	1	0
		ANNEX 2	First WHO global ministerial conference on “Ending tubeculosis in the sustainable development era: a multisectoral response”	1	0
WHA72	2020	ANNEX 5	Global action plan on promoting the health of refugees and migrants, 2019–2023	3	0
WHA73	2021	WHA73.1	COVID-19 Response	1	0
WHA74	2022	WHA74.14	Protecting, safeguarding and investing in the health and care workforce	2	0
		WHA74.16	Social determinants of health	1	0
		WHA74.17	Ending violence against children through health systems strengthening and multisectoral approaches	1	0
WHASS2	2022	NP	NP	NP	NP

* SP: the number of occurrences of the words “social protection”; * SA: the number of occurrences of the words “social assistance” or “cash transfer”; NP: Nothing particular.

**Table 2:** Agenda in World Health Assembly with Universal Health Coverage in the title and occurrence of words “social protection”.

WHA	Year	Agenda Number and Title	Document	SP *
WHA66	2013	17.3. Universal Health Coverage	A66/24	1
WHA67	2014	15.7. Health intervention and technology assessment in support of universal health coverage	A67/33	0
		15.8. Follow-up of the Recife Political Declaration on Human Resources for Health: renewed commitments towards universal health coverage	A67/34	0
WHA68	2015	17.1. Strengthening emergency and essential surgical care and anaesthesia as a component of universal health coverage	A68/31	0
WHA69	2016	17. Progress Report: F. Health intervention and technology assessment in support of universal health coverage	A69/43	0
WHA70	2017	17. Progress Report: K. Strengthening emergency and essential surgical care and anaesthesia as a component of universal health coverage	A70/38	0
WHA71	2018	NP		0
WHA72	2019	11.5. Universal health coverage. Primary health care towards universal health coverage	A72/12	0
		11.5. Universal health coverage. Community health workers delivering primary health care: opportunities and challenges	A72/13	0
		11.5. Universal health coverage. Preparation for the high-level meeting of the United Nations General Assembly on universal health coverage	A72/14	0
		12.9. Emergency and trauma care Emergency care systems for universal health coverage: ensuring timely care for the acutely ill and injured	A72/31	0
WHA73	2020	11.2. Follow-up to the high-level meetings of the United Nations General Assembly on health-related issues. Universal health coverage: moving together to build a healthier world	A73/4 EB146/6	0
WHA74	2021	33. Updates and future reporting. Emergency care systems for universal health coverage: ensuring timely care for the acutely ill and injured	A74/39	0
WHA75	2022	NP		0

* SP: the number of occurrences of the words “social protection”.

**Table 3:** Resolution/decisions in World Health Assembly with Universal Health Coverage in the title and occurrence of words “social protection”.

WHA	Year	Number	Title	SP *
WHA66	2013	WHA66.23	Transforming health workforce education in support of universal health coverage	0
WHA67	2014	WHA67.23	Health intervention and technology assessment in support of universal health coverage	0
		WHA67.24	Follow-up of the Recife Political Declaration on Human Resources for Health: renewed commitments towards universal health coverage	0
WHA68	2015	WHA68.15	Strengthening emergency and essential surgical care and anaesthesia as a component of universal health coverage	0
WHA69	2016	WHA69.1	Strengthening essential public health functions in support of the achievement of universal health coverage	0
WHA72	2019	WHA72.4	Preparation for the high-level meeting of the United Nations General Assembly on universal health coverage	0
		WHA72.16	Emergency care systems for universal health coverage: ensuring timely care for the acutely ill and injured	0

* SP: the number of occurrences of the words “social protection”.

## Data Availability

Not applicable.
